# A Capillary Electrophoresis-Based Method for the Measurement of Hydroxychloroquine and Its Active Metabolite Desethyl Hydroxychloroquine in Whole Blood in Patients with Rheumatoid Arthritis

**DOI:** 10.3390/molecules27123901

**Published:** 2022-06-17

**Authors:** Salvatore Sotgia, Angelo Zinellu, Nicola Mundula, Arduino A. Mangoni, Ciriaco Carru, Gian Luca Erre

**Affiliations:** 1 Department of Biomedical Sciences, School of Medicine, University of Sassari, 07100 Sassari, Italy; azinellu@uniss.it (A.Z.); carru@uniss.it (C.C.); 2 Rheumatology Unit, University Hospital Sassari (AOU-SS), 07100 Sassari, Italy; mundu@tiscali.it (N.M.); glerre@uniss.it (G.L.E.); 3 Flinders Medical Centre, Department of Clinical Pharmacology, College of Medicine and Public Health, Flinders University, Adelaide, SA 5042, Australia; arduino.mangoni@flinders.edu.au; 4 Department of Medicine, Surgery and Pharmacy, School of Medicine, University of Sassari, 07100 Sassari, Italy

**Keywords:** disease-modifying anti-rheumatic drug, autoimmune diseases, capillary electrophoresis, 4-aminoquinoline derivative, hydroxychloroquine, desethylhydroxychloroquine

## Abstract

A capillary electrophoresis method was developed to detect and measure hydroxychloroquine (HCQ) and its active metabolite desethyl hydroxychloroquine (DHCQ) in whole blood in patients with rheumatoid arthritis. The best separation in terms of peak area reproducibility, migration time, peak shape, and resolution of adjacent peaks was obtained in a 60 cm, 75 µm i.d. uncoated fused-silica capillary using a background electrolyte mixture of an aqueous 55 mmol/L TRIS solution brought to pH 2.6 with phosphoric acid and methanol (85:15) and a voltage and a temperature of separation of 20 kV and 30 °C, respectively. Analytes were separated in less than 12 min, with excellent linearity (R^2^ ≥ 0.999) in the concentration range of 0.5–8 µmol/L. The recovery of analytes spiked in whole blood was 99–101% for HCQ and 98–99% for DHCQ. Analysis of five samples from patients with rheumatoid arthritis receiving HCQ 400 mg daily yielded mean steady-state concentrations of 2.27 ± 1.61 and 1.54 ± 0.55 μmol/L for HCQ and DHCQ, respectively, with a HCQ to DHCQ ratio of 1.40 ± 0.77.

## 1. Introduction

Hydroxychloroquine (HCQ, (RS)-2-[4-(7-Chloro-4-quinolylamino)pentyl(ethyl)amino]-ethanol) is a 4-aminoquinoline derivative [1] used as a disease-modifying anti-rheumatic drug (DMARD) [2] to treat chronic inflammatory autoimmune diseases including systemic lupus erythematosus, sarcoidosis, Sjögren’s syndrome, and rheumatoid arthritis (RA), and, albeit less frequently, as antimalarial [3,4,5,6,7,8]. More recently, HCQ has also been used as a therapeutic option against COVID-19 infection [8] in view of its antiviral effects [9] and capacity to interfere with the glycosylation of SARS-CoV-2 cell receptors [10]. The daily dose ranges from 100 to 1200 mg, and after oral administration about 74% is absorbed within 4 h [11]. With a long terminal half-life of up to 40 days [12], therapeutic effects are typically delayed with steady-state concentrations achieved after around six months of treatment [13]. Although blood concentrations of HCQ rapidly increase after administration, they also decrease rapidly following extensive trapping by lysosome-rich tissues such as the liver [14,15]. In the liver parenchyma, HCQ is also metabolized to different products, including the primary active metabolite desethyl hydroxychloroquine (DHCQ) [16]. The reported blood-to-plasma ratio is 7.2 [17], and the concentrations measured in whole blood are less variable than those in plasma, where HCQ is also partially bound to proteins [18]. Importantly, due to the extensive accumulation of HCQ in blood cells, especially white cells, plasma concentrations can be significantly affected by cell lysis during sample processing [17]. Thus, the measurement of HCQ concentrations in whole blood is preferable for pharmacokinetic studies, monitoring patient compliance, and optimizing treatment efficacy [18]. Analytical methods based on hyphenated gas chromatography–mass spectrometry [19,20] and HPLC coupled to different detectors, including fluorimeter, spectrophotometer, and mass spectrometry, have been extensively used for quantitative analysis of HCQ in tissues and different biological fluids, e.g., plasma, whole blood, and urine [21,22,23,24]. Stand-alone spectrophotometric methods have also been used to measure HCQ concentrations in tablets, raw materials, bulk drugs, and different formulations [25,26,27]. By contrast, capillary electrophoresis (CE) has been used infrequently to analyze HCQ and its metabolites [11]. Consequently, published methods are limited to the measurement of HCQ and its metabolites in the microsomal fraction of liver homogenates or for its enantioselective analysis [28,29]. Regardless of the technique used, however, currently available methods are labor-intensive and not practical, e.g., they involve time-consuming and cumbersome sample preparation such as extraction by liquid–liquid partition or evaporation to dryness under vacuum, thus limiting their routine use in clinical practice. To minimize sample pre-treatment and exploit the well-documented advantages of CE over HPLC in terms of reduced operating costs, simplicity, environmental impact, high separation efficiency, and short analysis times [30,31,32], this study describes the development of an original CE method for the rapid measurement of HCQ and its active metabolite DHCQ in whole blood in patients with RA.

## 2. Materials and Methods

### 2.1. Chemicals and Reagents

Acetonitrile (ACN) HPLC grade, methanol (MeOH), ethanol (EtOH), Trizma Base (TRIS), Trizma hydrochloride pH 3.5–5.0 (TRISH), phosphoric acid (H_3_PO_4_), sodium hydroxide (NaOH), hydrochloric acid (HCl), dimethyl sulfoxide (DMSO), trichloroacetic acid (TCA), metaphosphoric acid (MPA), and 5-sulfosalicylic acid hydrate (SSA) were ordered from Merck Italia (Milan, Italy). Hydroxychloroquine sulfate (HCQ) and desethyl hydroxychloroquine (DHCQ) were purchased from CABRU sas (Milan, Italy), a licensed dealer of Cayman Chemical Company (Ann Arbor, MI, USA). High-purity water used throughout the experiments was obtained with a Millipore Milli-Q system.

### 2.2. Apparatus and CE Conditions

An Agilent 7100 CE system equipped with a diode array detector and the ChemStation software (Revision C.01.03) for instrument control and data analysis (Agilent Technologies, Milan, Italy) was used. CE was performed on an uncoated fused-silica capillary (Agilent Technologies, Milan, Italy) measuring 75 µm i.d. × 60 cm total length. Before the separation, at the beginning of each experimental session, the capillary was flushed with, in order, 100 mmol/L NaOH, 100 mmol/L HCl, and BGE for 10 min each at 940 mbar. Quantitative CE analysis was performed in normal mode (anode at the CE inlet and cathode at the outlet side) at +20 kV, injecting sample mixture hydrodynamically at 100 mbar × 10 s. During analysis, the capillary was thermostated at 30 °C, and, between analyses, the capillary was rinsed with 100 mmol/L NaOH and 100 mmol/L HCl for 60 s each at 940 mbar, then filled with BGE for 90 s at 940 mbar. Detection was performed at wavelengths of 220, 236, 256, 329, and 343 nm, with the former used for the quantitative analysis of analytes and to plot the presented electropherograms.

### 2.3. Participants in Study and Sample Collection

Five RA female patients aged 60 years or older receiving a daily HCQ dose of 400 mg at steady state were randomly selected from the outpatient clinics of the Rheumatology Unit, University Hospital Sassari (AOU-SS), Sassari. After informed written consent was given, fasting blood samples were obtained in the morning by 09:00 a.m., after an approximately ten-hour overnight fast. Blood was collected by venipuncture in 5 mL EDTA vacutainer tubes. Whole blood was rapidly stored in 300 µL aliquots at −80 °C until use. The study was performed following the principles outlined in the Declaration of Helsinki, and all procedures were approved by the ethics committee of the Azienda ASL 1 of Sassari, Italy (Bio-RA study, 2219/CE-2015).

### 2.4. Sample Treatment

To lyse blood cells and precipitate the proteins, 100 µL of TCA 4% *w*/*v* (final concentration) was added with a 300 µL volume of whole blood and mixed thoroughly by vigorous vortex-mixing. Samples were sonicated for 5 min to break up clumps, then centrifuged at 17,000× *g* for 10 min at room temperature. Finally, 120 µL of the clear supernatant was recovered for analysis.

### 2.5. Solutions

Stock solutions of HCQ and DHCQ in DMSO were prepared at a concentration of 1.15 mmol/L and 1.43 mmol/L, respectively, and stored at −20 °C until use. Solutions of TCA, MPA, and SSA, tried as lysis and protein precipitation agents, were prepared as 50% *w*/*v* stock solutions in ultrapure water, and diluted appropriately to reach a final concentration in the sample of 4% *w*/*v*. Buffer solutions of TRIS and TRISH, with concentrations ranging between 35 and 100 mmol/L and pH between 2 and 4.5, were tried as BGEs. Within this concentration range, TRIS buffers were brought to the desired pH values with H_3_PO_4_, while the TRISH solutions were used without adjusting the pH, at the native pH values of around 4.5. Rinse solutions of NaOH and HCl used to wash the capillary were prepared in ultrapure water at a concentration of 100 mmol/L.

## 3. Results

### 3.1. Sample Treatment

Different acids, i.e., TCA, SSA, and MPA, and organic solvents, i.e., ACN and MeOH, were evaluated as blood cell lysis and protein precipitation agents. In all cases, after adding the reagents, whole blood samples were sonicated for 5 min. To minimize the dilution of the samples, ACN and MeOH were added to the sample in a 1:1 solvent-to-whole blood ratio. For the same reason, a minimum volume of acid was added to the sample to reach a final concentration of 4% *w*/*v*. Despite a slight increase of background noise using SSA, the effect of TCA and SSA was essentially equivalent, giving after centrifugation a clear supernatant and a similar electrophoretic pattern without interfering with the detection of the analytes. In contrast, MPA and MeOH yielded a dark-red supernatant because of the incomplete lysis of blood cells and partial protein precipitation, preventing the analysis by CE. Conversely, with a pale-yellow supernatant and a reddish hue, ACN did not prevent the analysis by CE, despite the current frequently dropping to zero during the run. On these bases, TCA at a final concentration in the sample of 4% *w*/*v* was chosen as the preferred lysis and precipitating agent.

### 3.2. Electrophoretic Conditions

**Figure 1:**
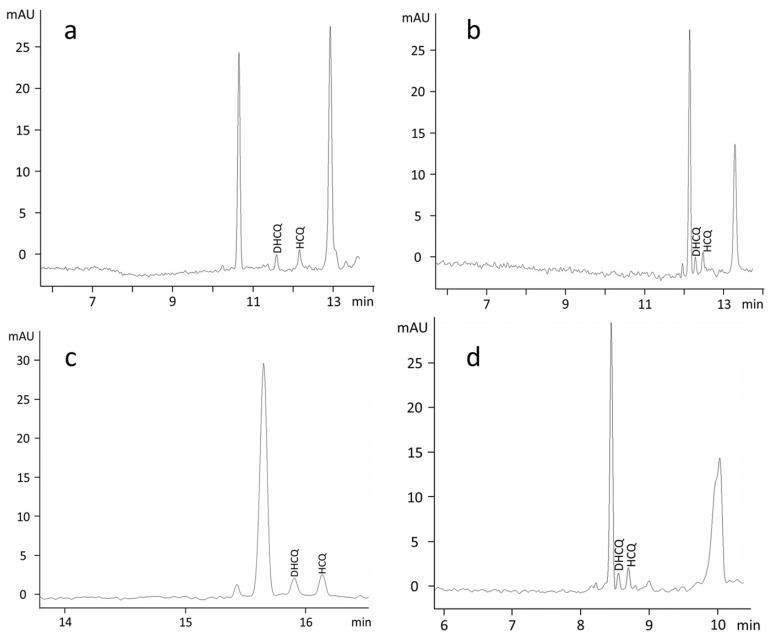
Electropherograms of a clinical sample obtained using as a BGE, (**a**) a 100 mmol/L TRISH solution at pH 4.5 and a separation voltage of 15 kV at a temperature of 30 °C, (**b**) a 100 mmol/L TRIS solution brought to pH 4.5 with H_3_PO_4_ and a separation voltage of 15 kV at a temperature of 30 °C, (**c**) a 55 mmol/L TRIS solution brought to pH 2.6 with H_3_PO_4_ and a separation voltage of 15 kV at a temperature of 30 °C, and (**d**) a 55 mmol/L TRIS solution brought to pH 2.6 with H_3_PO_4_ and a separation voltage of 20 kV at a temperature of 30 °C.

**Figure 2:**
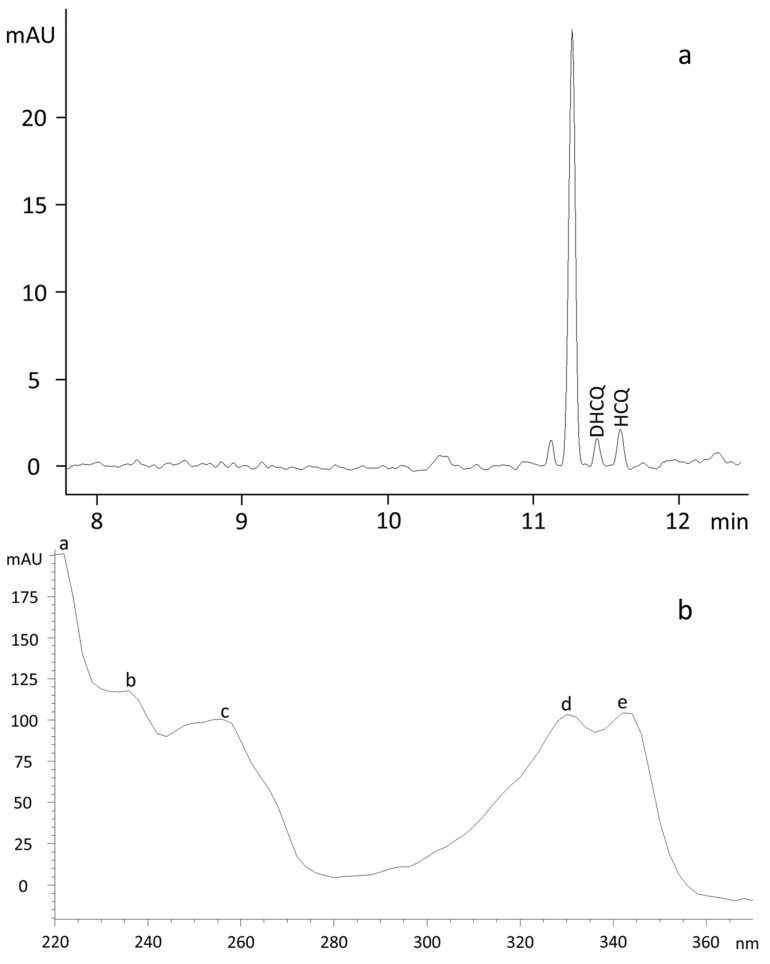
(**a**) Electropherogram of a clinical sample obtained using as a BGE, (**a**) a mixture of an aqueous 55 mmol/L TRIS solution brought to pH 2.6 with H_3_PO_4_ and MeOH (85:15), and a voltage and a temperature of separation of 30 °C and 20 kV and (**b**) UV-Vis spectra of analytes recorded in the range of 190–400 nm with maximum absorbance at (a) 220, (b) 236, (c) 256, (d) 329, (e) and 343 nm.

As shown in Figure 1a, the separation obtained using a 100 mmol/L TRISH solution at pH 4.5 as a BGE and a separation voltage of 15 kV at a temperature of 30 °C was adequate in terms of resolution between the analytes. However, with a percentage relative standard deviation (%RSD) of 12%RSD (*n* = 5) for DHCQ and 11%RSD (*n* = 5) for HCQ, the corrected peak area reproducibility proved to be quite variable from run to run. Compared to the peak area, peak retention time reproducibility was higher, below 1.5%RSD for both analytes, and was even better (<0.4%RSD) using a TRISH 55 mmol/L solution. However, although at this concentration the peak area reproducibility of HCQ improved (5%RSD, *n* = 5), it worsened for DHCQ (15%RSD, *n* = 5). The further reduction of the TRISH concentration did not improve the reproducibility of the areas but led to a gradual deterioration in resolution between the analytes. Moreover, no changes were observed in the reproducibility by lowering the pH to a more acidic environment or replenishing the BGE after every run. As shown in Figure 1b, the replacement of TRISH with a TRIS solution at the same concentration and pH value (100 mmol/L, pH 4.5), leaving unchanged the separation conditions of temperature and voltage (30 °C, 15 kV), resulted in a worsening of resolution between the analytes. Although the peak area reproducibility was improved for DHCQ (9%RSD, *n* = 5), variability was still high for HCQ (14%RSD, *n* = 5). Moreover, the reduction of the concentration of TRIS to 55 mmol/L keeping the pH at 4.5 did not produce any significant changes. The lowering of the pH of the 55 mmol/L TRIS solution to 2.6 produced a marked increase in migration times of about 33% (Figure 1c). In such conditions, the resolution between analytes improved, despite some variability in the reproducibility of the areas, on average around 10%RSD. The rise of separation voltage from 15 to 20 kV resulted in the resolution of analytes in less than 9 min with a loss of resolution between analytes and no enhancement of peak area reproducibility (Figure 1d). As shown in Figure 2a, the addition of MeOH as an organic modifier to the running buffer allowed one to slightly increase the migration time of analytes resulting in an improvement of resolution and of peak area reproducibility (<8%RSD). By contrast, no improvements were obtained using other organic modifiers such as ACN and EtOH. Consistent with the literature, under the operative condition, the absorbance spectra of HCQ and DHCQ recorded in the 190–400 nm range showed maximum absorbances at wavelengths of 220, 236, 256, 330, and 342 nm with the latter highly specific for the detection of the analytes as it is associated with the quinolinium group (Figure 2b). However, to increase the chance of detection, analyses were performed at the maximum absorbance wavelength of 220 nm.

### 3.3. Linearity, Accuracy, Precision and Sensitivity

To construct the calibration curves, analytes at known scalar concentrations were added to drug-free whole blood obtained from healthy volunteers. The ordinary least-squares method was used to fit the calibration curves and to find a range of concentrations with a linear correlation between peak area and concentration. As showed in Table 1, with a coefficient of determination (R^2^) of 0.999 (N = 5), linearity was found for both analytes over a concentration range of 0.5–8 µmol/L that, based on the concentrations observed in previous studies, adequately covers the analytical range of interest. Thus, seven concentrations in such a range were used for quantification analyses. Three quality control (QC) samples, prepared at three concentration levels of 0.6, 3.2, and 7.0 µmol/L for low (LoQC), medium (MeQC), and high (HiQC) quality control, respectively, were used to assess intra- and inter-day precision. Four replicates for each QC were performed and %RSDs for intra-day precision for both analytes were around 4%. Similarly, %RSDs for inter-day precision for HCQ and DHCQ measurements were below 6%. Accuracy was assessed by determination of the recovery of the method at three different concentrations by spiking known amounts of standards to real samples. The mean recovery of HCQ and DHCQ was between 99% and 101% and between 98% and 99%, respectively. Based on the standard deviation of the response and the slope, the limit of detection (LOD) and the limit of quantification (LOQ) were determined as the smallest signals able to provide peaks with a signal-to-noise ratio of 3 and 10, respectively. LOD and LOQ were 0.13 ± 0.04 and 0.37 ± 0.03 µmol/L for HCQ and 0.13 ± 0.06 and 0.43 ± 0.04 µmol/L for DHCQ.

### 3.4. Clinical Application

HCQ and DHCQ were detectable in all the whole blood samples from RA patients receiving HCQ 400 mg/day. The mean steady-state concentrations were 2.27 ± 1.61 and 1.54 ± 0.55 μmol/L for HCQ and DHCQ, respectively.

## 4. Discussion

**Figure 3:**
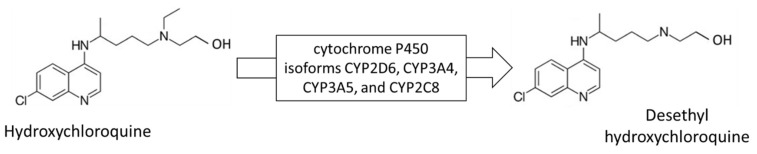
Structures of hydroxychloroquine and desethyl hydroxychloroquine.

HCQ and DHCQ are amphiphilic weakly basic compounds with three potentially protonable nitrogen atoms (Figure 3).

The nitrogens of HCQ show pKa of 4.0, 8.3, and 9.7 [33], and those with the highest values undergo protonation under physiological conditions [14]. The pKa values of DHCQ are unknown, but given the close structural resemblance with HCQ, it is conceivable that its nitrogens behave similarly. Accordingly, performing the CE at a pH > 4 would benefit from both the electroosmotic flow (EOF) effect and the cathodic attraction due to the positive charge of analytes. Thus, a 100 mmol/L TRISH solution at its native pH of 4.5 used as a BGE and a voltage and temperature separation of 15 kV and 30 °C, respectively, led to the quick separation of the analytes as both EOF and the positive electrical charge drive the analytes towards the cathode (Figure 1a). However, despite a slight improvement obtained by reducing the concentration of the buffer to 55 mmol/L, the buffering capacity of TRISH was exhausted within a few runs, resulting in a marked variability of peak areas and the need for continuous renewal of the buffer. The replacement of TRISH with a 100 mmol/L TRIS solution brought to pH 4.5 with H_3_PO_4_ showed more stability. However, the synergistic action between EOF and electrophoretic mobility impairs the resolution of adjacent peaks affecting the reproducibility of the peak areas negatively (Figure 1b). Unlike TRISH, reducing buffer concentration to 55 mmol/L did not improve the separation. Notwithstanding, to gain advantages from the decreased Joule heating resulting from the current flow through the electrolyte, this buffer concentration was used anyway to conduct the subsequent experiments. However, given the unsatisfactory effect resulting from the combination of electrical attraction and EOF, the TRIS buffer was trialed at pH values < 4 to suppress or limit the impact of EOF. Uncoated fused-silica capillaries exhibit EOF toward the cathode, and its magnitude drops with decreasing pH [34]. On the other hand, at a pH < 4, the analytes can be expected to be in the full protonation state (3+) and, therefore, to have the highest positive charge density and experience the maximum cathodic attraction [35]. As expected, using as a BGE a 55 mmol/L TRIS solution brought to pH 2.6 with H_3_PO_4_ leaving unchanged the separation conditions of temperature and voltage, the migration time of analytes increased markedly (about 33%) due to the lack of EOF contribution to the movement of the analytes (Figure 1c). As a result, the reproducibility of peak areas improved with the increased resolution between adjacent peaks. Attempts to accelerate the CE by changing the voltage in the range of from 15 to 20 kV were unsuccessful as both resolution and reproducibility were impaired with increasing voltage (Figure 1c). Similarly, no noteworthy results were obtained by modifying the separation temperature. Therefore, in an attempt to shorten the analysis time while retaining a good resolution, BGE was added with different organic modifiers such as EtOH, ACN, and MeOH, as the latter may influence the mobilities of analytes, affecting both the EOF and their electrophoretic mobility [36]. The addition of both EtOH and ACN did not enable a satisfactory resolution/separation compared to the TRIS buffer alone. By contrast, the addition of MeOH led to a slight increase in the migration times of the analytes, resulting in a better resolution of the adjacent peaks and an improvement of the variability of peak areas. Thus, a satisfactory balance between peak area reproducibility, migration time, peak shape, and resolution of adjacent peaks was obtained using as a BGE a mixture of an aqueous 55 mmol/L TRIS solution brought to pH 2.6 with H_3_PO_4_ and MeOH (85:15), and a voltage and a temperature of separation of 20 kV and 30 °C, respectively. Under these optimized conditions, analytes were baseline separated in less than 12 min (Figure 2a). Applying the established analytical method to determine the analytes in five RA patients treated with a stable dose of HCQ 400 mg/day yielded mean steady-state concentrations of 2.27 ± 1.61 and 1.54 ± 0.55 μmol/L for HCQ and DHCQ, respectively. Although the ratio of HCQ to DHCQ of 1.40 ± 0.77 was slightly lower than that reported by others, 1.75 ± 0.37 [37], our findings are in line with previous observations. The coefficients of variation (CV%) of 36 and 71% for HCQ and DHCQ, respectively, confirm the high inter-individual variability observed by other authors [38]. With DHCQ being a metabolite of HCQ, the relatively higher CV% values could signal a high inter-individual variability in metabolic rate [16].

## 5. Conclusions

We developed a robust method for rapid determination of HCQ and DHCQ in whole blood using CE. Sample preparation was simple, inexpensive, and only required blood cell lysis and removal of proteins by an acidic treatment with TCA. Overall, given the short sample preparation and electrophoresis run time, the method may be suitable for the fast and reproducible quantitative determination of the analytes in large clinical trials and routine analysis for pharmacokinetics studies and monitoring patient compliance and optimizing treatment efficacy.

## Figures and Tables

**Table 1:** Summary of validation parameters of the assay. Concentrations for LoQC, MeQC, and HiQC were 0.6, 3.2, and 7.0 µmol/L, respectively.

Analyte	Linear Range (µmol/L)	LOD (µmol/L)	LOQ (µmol/L)	Intra-Day Precision (%RSD)		Inter-Day Precision (%RSD)		Recovery (%)
HCQ	0.5–8	0.13 ± 0.04	0.37 ± 0.03	LoQC	4.4	LoQC	7.1	99–101
				MeQC	2.8	MeQC	5.2	
				HiQC	5.0	HiQC	4.9	
DHCQ	0.5–8	0.13 ± 0.06	0.43 ± 0.04	LoQC	5.1	LoQC	5.1	98–99
				MeQC	3.2	MeQC	5.2	
				HiQC	4.3	HiQC	6.9	

## Data Availability

Not applicable.

## Sample Availability

Samples of the compounds are not available from the authors.
